# Endoplasmic Reticulum Stress in Elderly Patients with COVID-19: Potential of Melatonin Treatment

**DOI:** 10.3390/v15010156

**Published:** 2023-01-04

**Authors:** Giou-Teng Yiang, Chia-Chao Wu, Chien-Lin Lu, Wan-Chung Hu, Yi-Ju Tsai, Yiao-Mien Huang, Wen-Lin Su, Kuo-Cheng Lu

**Affiliations:** 1 Department of Emergency Medicine, Taipei Tzu Chi Hospital, Buddhist Tzu Chi Medical Foundation, New Taipei 231, Taiwan; 2 School of Medicine, Tzu Chi University, Hualien 970, Taiwan; 3 Division of Nephrology, Department of Internal Medicine, Tri-Service General Hospital, National Defense Medical Center, Taipei 114, Taiwan; 4 Department and Graduate Institute of Microbiology and Immunology, National Defense Medical Center, Taipei 114, Taiwan; 5 Division of Nephrology, Department of Medicine, Fu Jen Catholic University Hospital, School of Medicine, Fu Jen Catholic University, New Taipei 24352, Taiwan; 6 Department of Clinical Pathology, Taipei Tzu Chi Hospital, Buddhist Tzu Chi Medical Foundation, New Taipei 231, Taiwan; 7 Graduate Institute of Biomedical and Pharmaceutical Science, College of Medicine, Fu Jen Catholic University, New Taipei 243, Taiwan; 8 Department of Dentistry, Taipei Tzu Chi Hospital, Buddhist Tzu Chi Medical Foundation, New Taipei 231, Taiwan; 9 Division of Pulmonary and Critical Care Medicine, Department of Internal Medicine, Taipei Tzu Chi Hospital, Buddhist Tzu Chi Medical Foundation, New Taipei 231, Taiwan; 10 Division of Nephrology, Department of Medicine, Taipei Tzu Chi Hospital, Buddhist Tzu Chi Medical Foundation, New Taipei 231, Taiwan

**Keywords:** melatonin, COVID-19, aging, ER stress, unfolded protein response, SARS-CoV-2

## Abstract

Aging processes, including immunosenescence, inflammation, inflammasome formation, genomic instability, telomeric attrition, and altered autophagy, are involved in viral infections and they may contribute to increased pathophysiological responses to the SARS-CoV-2 infection in the elderly; this poses additional risks of accelerated aging, which could be found even after recovery. Aging is associated with oxidative damage. Moreover, SARS-CoV-2 infections may increase the production of reactive oxygen species and such infections will disturb the Ca^++^ balance via an endoplasmic reticulum (ER) stress-mediated unfolded protein response. Although vaccine development and anti-inflammation therapy lower the severity of COVID-19, the prevalence and mortality rates are still alarming in some countries worldwide. In this review, we describe the involvement of viral proteins in activating ER stress transducers and their downstream signals and in inducing inflammation and inflammasome formation. Furthermore, we propose the potential of melatonin as an ER stress modulator, owing to its antioxidant, anti-inflammatory, and immunoregulatory effects in viral infections. Considering its strong safety profile, we suggest that additive melatonin supplementation in the elderly could be beneficial in treating COVID-19.

## 1. Introduction

Coronavirus disease 2019 (COVID-19) is caused by the severe acute respiratory syndrome coronavirus 2 (SARS-CoV-2). Older adults with comorbidities and people with chronic medical conditions and compromised immune systems are at risk of more severe COVID-19 [1]. Even though SARS-CoV-2 is highly virulent, its clinical course and outcome vary significantly across individuals and populations. Host–microbe interactions have been considered to mediate the broad spectrum of clinical presentations, including pneumonia, liver and kidney dysfunction, cardiovascular illness, cerebrovascular thromboembolism events, and mortality [2].

The aging process is characterized by a progressive decline in the effectiveness and ability of a cell to deal with external and internal stress, resulting in the development of aging disorders. The hallmarks of aging and the interactions between the host, the virus, and the environment are proposed to influence health outcomes in older adults through immune system regulation [3,4]. Aging increases the morbidity and mortality rates for several diseases [5]. Immunosenescence, defined as immune system deterioration due to aging, has attracted much attention, owing to its contribution to the development of infectious diseases, autoimmune dysregulation, and cancer in the elderly [6,7].

The endoplasmic reticulum (ER) physiologically participates in protein synthesis, folding, modification, and secretion, thus playing a foundational role in cellular function [8]. The stress response induced by overwhelming misfolded and/or unfolded protein accumulation in the ER lumen is named the unfolded protein response (UPR). The UPR triggers a series of signaling and transcriptional actions that rescue ER homeostasis by attenuating protein translation, enhancing the digestion of misfolded proteins, and accentuating the production of chaperones that participate in protein folding. If ER homeostasis fails to be restored, then the UPR progresses to apoptosis [9]. ER stress is invoked in innate immune signaling against invading pathogenic organisms, and dysregulated UPR signaling is reportedly involved in several infectious diseases [10]. Viruses have evolved a diversified mechanism for evading host immune responses. Recent studies have demonstrated that viruses may perturb virus–host UPR interactions and establish a favorable environment for persistent infection [11].

The activity of autophagy processes is known to be reduced by aging. Compromised autophagy predisposes to viral infection because the accumulation of damaged organelles and protein aggregates can impair viral degradation and dysregulate innate and adaptive immunity [12]. Although certain viruses can successfully antagonize autophagy functions, autophagy-induced immune responses can still cause tissue disruption. Therefore, the age-induced dysregulation of autophagy may be related to the severity of clinical symptoms and the devastating prognosis thereof in elderly patients with COVID-19.

Despite the tremendous success of vaccines in controlling the SARS-CoV-2 infection and the limited number of RNA virus-specific drugs in development and clinical use, the urgent need for new antiviral medication against emerging RNA viruses remains crucial. The natural compound thapsigargin is reported to combat the coronavirus infection by targeting the enhancement of host cell function, thereby eliciting chemical ER stress [13,14]. However, most nutraceutical compounds used in patients with COVID-19 induce the suppression of ER stress. Nutraceutical melatonin (N-acetyl-5-methoxytryptamine) can be produced in various tissues in the body. However, the pineal gland, associated with circadian rhythms, is a prominent cause of decline with age; therefore, pineal melatonin is prominently featured in all aging theories [15,16]. Furthermore, recent studies have demonstrated that melatonin participates in several physiological functions and exerts immunomodulatory and antioxidant effects [17], suggesting its potential in treating COVID-19.

Because the prevalence and mortality rates are still alarming in some countries, further additive therapy for COVID-19 is urgent. Therefore, this review article details the involvement of aging-related processes, ER stress, and the downstream pathways thereof, including UPR, inflammation, and autophagy in SARS-CoV-2 infection. Furthermore, we elaborate on the possible therapeutic effects of melatonin in elderly patients with COVID-19, with a particular focus on ER stress.

## 2. Aging and Immune Response in COVID-19

The silent transmission of SARS-CoV-2 during asymptomatic or presymptomatic stages, especially in the elderly, is likely to cause serious complications owing to underlying chronic illnesses. High-risk patients with COVID-19, including the elderly, those who are chronically ill, and obese individuals, are more likely to experience neurological or cardiovascular complications because of interactions between the SARS-CoV-2-specific envelope protein and its corresponding host cell receptor [18,19,20]. Therefore, exploring the mechanisms underlying the host response to viral infection among older patients is worthy of investigation [21].

### 2.1. Signs of Aging and COVID-19

Normal aging processes occur from early adulthood and are involved in the frailty, morbidity, and mortality of older adults [22]. Aging is characterized by increased genomic instability, obvious telomere attrition, mitochondrial dysfunction, prominent cellular senescence and immunosenescence, inflammaging and inflammasome activation, and deficient autophagy. The main mechanism underlying the aging process and aging-related diseases is chronic inflammation. These changes reduce the adaptive capacity of cells to cope with stress as they age and contribute to stress intolerance, which interferes with normal cellular and systemic functions; these changes are also associated with chronic diseases [23] and influence viral infection directly or indirectly.

### 2.2. Immune Reactions Elicited by SARS-CoV-2: Immunosenescence and Inflammation in Aging

SARS-CoV-2 holds a single-stranded RNA (ssRNA) genome that encodes the essential structural proteins to produce viral particles. Within infected host cells, the viral structural proteins such as S (spike), M (membrane), and E (envelope) proteins will insert into the host ER membrane and are then transported onto the ER–Golgi intermediate compartment (ERGIC) to form the virion [24]. The most abundant glycoprotein in the viral particle is the M protein, which helps with virus assembly. The S protein plays a key role in viral pathogenicity, infectivity, and possible mutation because of its receptor recognition capability and cell membrane fusion ability [25]. The S protein comprises two subunits: the S1 subunit has a receptor-binding domain that recognizes and binds to the host angiotensin converting enzyme 2 (ACE2) receptor; the S2 subunit is responsible for viral and host cell membrane fusion that facilitates viral entry [26]. The N protein is an RNA-binding protein that packages the viral genome and efficiently interacts with the M protein during virion assembly [27]. Comparative analysis of the SARS-CoV-1 and SARS-CoV-2 genome sequences revealed high similarity in amino acid residues in the S protein, as well as in the N, M, and open reading frame (ORF) 3a proteins, suggesting that these viruses have similar pathogenic roles [28].

ACE2 can convert angiotensin-II into angiotensin-(1–7). However, the cell membrane-bound ACE2 can be used by the SARS-CoV-2 S protein as a binding receptor and can be utilized for host cell entry. ACE2 is highly expressed in nasopharyngeal, lung, vascular endothelial, renal tubular, and small intestine epithelial cells, as well as activated type 1 macrophages, including alveolar macrophages, which are a direct target of SARS-CoV-2 [29,30]. Recent studies have shown that the host neuropilin-1 receptor protein promotes SARS-CoV-2 infection through furin-accelerated partial degradation and separation of the S1 and S2 domains of the spike protein. After cleavage of the S protein, neuropilin-1 favors binding to the RRAR amino acid motif of the S1 domain and facilitates viral attachment to host cell membrane [31,32]. Upon entry into the host cell, the SARS-CoV-2 genome could be replicated by host cell RNA polymerase and the host translational machinery could be used to generate viral proteins in the host cell cytoplasm. After adequate viral assembly, the budding of virions subsequently occurs at the host ERGIC and mature virions could be released from infected host cells by secretory machinery in smooth-walled vesicles; this process ultimately results in endoplasmic reticulum stress [3,33].

Pattern recognition receptors (PRRs) are one kind of host sensor proteins that are mainly expressed by innate immune effector cells to directly recognize specific molecules on the pathogen surface, which are known as pathogen-associated molecular patterns (PAMPs) or damage-associated molecular patterns (DAMPs). The PAMPs are small molecules with conserved motifs and are associated with lipopolysaccharides from Gram-negative bacteria, whereas DAMPs are endogenous components released from the damaged or dying host cell in response to cell stress [34,35]. Based on protein domain homology, PRRs can be classified as (1) absent in melanoma-2 (AIM2)-like receptors (ALRs), (2) nucleotide-binding oligomerization domain (NOD)-like receptors (NLRs), (3) C-type lectin receptors (CLRs), (4) retinoic acid-inducible gene I (RIG-I)-like receptors (RLRs), and (5) toll-like receptors (TLRs) [36].

TLRs are predominantly expressed intracellularly and on the cell membrane surface, whereas RLRs and NLRs present in the cytoplasm [37]. Among the human TLR family, TLR3, TLR7, and TLR8 reside in the endosome compartment and can act as endosomal RNA sensors [38]. TLR3 mainly senses double-stranded viral RNA (dsRNA), including the genome of dsRNA viruses and intermediates produced during ssRNA replication, whereas TLR7 and TLR8 sense viral ssRNA and bacterial RNA [39,40]. TLR9 specifically recognizes cytosine–phosphate–guanine (CpG) motifs in the DNA of microbes and viruses [41].

**Figure 1:**
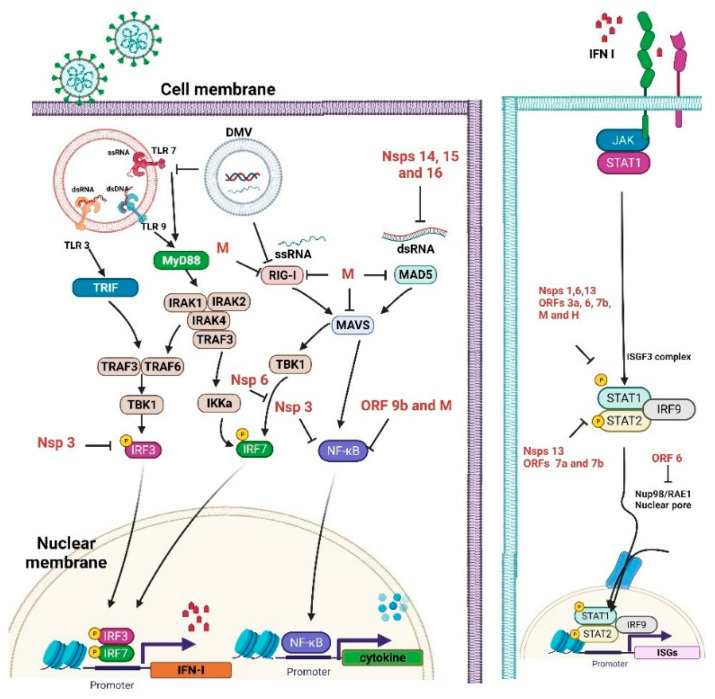
Immune evasion by severe acute respiratory syndrome coronavirus 2 (SARS-CoV-2).

In the setting of the COVID-19 infection, TLR3, TLR7, TLR8, and TLR9 are likely relevant as the key gatekeepers in detecting and combating viral infections. After recognition of endogenous DAMPs, TLRs will recruit intracytoplasmic toll-interleukin-1 receptor (TIR) domain containing adaptor proteins to initiate intracellular signal transduction and increase the production of proinflammatory cytokines and chemokines, augment type I interferons (IFN) production, and release from immune cells to fight against viral infection [42]. Thereafter, IFNs induce IFN-stimulated genes (ISGs) to mediate antiviral effects, including interference with viral entry, limiting viral transcription/replication, and inhibiting virion formation and budding [43,44]. IFNs can also directly inhibit viral shedding, block viral transmission to the next host cell, and augment the immune system by promoting the activity of different immune cells [45]. SARS-CoV-2 has been suggested to suppress type I IFN production, antagonize IFN downstream signaling, and inhibit the ISG response, thus perturbing the immune system [46,47] (Figure 1).

Unlike TLRs, which act as endosomal sensors, RLRs have been extensively studied as cytosolic dsRNA virus sensors to mediate IFN transcription and activate mitochondrial antiviral-signaling (MAVS) proteins [48]. Upon viral infection, RIG-I interacts with MAVS proteins to activate latent NF-κB and interferon regulatory factors (IRF)3/7, subsequently inducing proinflammatory cytokine production [49].

Inflammasomes are intracellular multiprotein complexes in the cytoplasmic compartment and are composed of NLR protein 3 (NLRP3), adaptor apoptosis-associated speck-like (ASC) protein, and procaspase-1. Once activated, inflammasome formation initiates the expression of the caspase-1 protease and promotes proinflammatory cytokine secretion [50,51]. The NLRP3 inflammasome assembly needs two checkpoints: the priming step and the activation step. Priming is essential for NLRP3-mediated gene expression and involves post-translational modification to form the correct conformation, enabling inflammasome assembly. Upon inflammasome activation in response to viral particles, the NLRP3 will polymerize to ASC and then recruit procaspase-1 to cleave pro-IL-1β into IL-1β combined with the production of pro-IL-18. Inflammaging is an aging process that describes the low-grade inflammation associated with age-related chronic diseases. Viral infection can stimulate NLRP3 inflammasome activity, thereby aggravating immunological responses that decrease upon aging [52].

Immunosenescence refers to the gradual decline in immune system function during aging. Diminished innate immune responses upon aging reduce the type I IFN reaction to viral infection (e.g., influenza) in neutrophils, macrophages, monocytes, and NK cells [53,54]. Furthermore, costimulatory signals on antigen-presenting cells, which are needed for optimal T cell responses, are reduced on macrophages, B cells, and NK cells [55].

Overproduction of proinflammatory cytokines can occur through the activation of both the NF-κB pathway and NLRP3 inflammasome, also called a cytokine storm, which can cause multiorgan injury in COVID-19 [56,57]. Notably, the SARS-CoV-2 infection also increases the expression of A disintegrin and metalloprotease 17 (ADAM17), also called TNF-α-converting enzyme (TACE), which is a protease involved in processing TNF-α and TNF receptors (TNFRs) and the shedding of cell membrane ACE2 [58]. ACE2 can convert angiotensin II into angiotensin-(1–7), which possesses anti-inflammatory, antioxidant, and antifibrotic properties [59].

The amount of naïve T cells that enable antigen-presenting cells to communicate with antigen-specific CD_8_^+^ T cells decreases upon aging [60,61]. Notably, the less potent immune responses observed during aging have been demonstrated to be influenced by sex chromosome genes and sex hormones, resulting in differential vulnerability to viral infection and vaccination efficacy between the sexes [62]. Sex- and age-related epigenetic and possible transcriptomic discrepancies in immune cells can also result in age-dependent sex differences that influence the severity of COVID-19 [63].

As illustrated in Figure 1, SARS-CoV-2 proteins can negatively control various immune processes, including IFN production and signaling, ISGs, and pathogen recognition [64]. The viral proteins may inhibit various host signaling cascades and the viral RNA may play an important role in this process. The viral RNA may be capped with guanosine and then methylated at the 5’ end via the influence of SARS-CoV-2 non-structural proteins, resulting in the efficient escape of viral dsRNA from host cell sensor recognition [65]. Some viral proteins also decrease the activities of intermediaries of the IFN signaling cascade. The viral ORF9b affects the MAVS inside mitochondria, resulting in a decrease in TRAF6 and TRAF3. The viral nsp13 and nsp15 proteins can interact with TBK-1 and positively modulate IRF3 [66]. Nsp1 is also an important virulence factor that negatively modulates general gene expression in the host cell, thereby preventing the innate immune system from defending against the viral infection [67]. Nsp3 also negatively impacts IFN signaling through a reversible post-translational modification of ISG15 [68].

### 2.3. Genomic Instability and Telomere Attrition

As previously mentioned, the immune system becomes less effective with aging. Mutation accumulation in somatic DNA, genomic instability, and impaired DNA repair all contribute to the aging process in immune cells [69]. Oxidative stress to DNA has been linked with the pathological changes associated with age. Moreover, as a promoter of DNA repair, p53 acts as a critical factor in responding to low-grade oxidative stress and protecting cells against oxidative injury. High-grade oxidative stress, such as virus infection-related inflammation, leads to sustained p53 activity and increased permeability of the outer mitochondrial membrane, ultimately resulting in apoptosis [70]. Although p53 may downmodulate CoV replication by regulating the cell cycle [71], CoV papain-like proteases degrade p53, allowing infected cells to replicate [72]. Many RNA viruses can damage the host cell DNA during their life cycle, induce genetic instability, and manipulate the DNA damage response (DDR) machinery for detecting and repairing DNA damage. The host DNA damage response elicited in an RNA virus infection can play a role in inducing host cell apoptosis, somatic mutations, and overstimulating inflammatory responses [73].

Severe T-cell lymphopenia is a clinical characteristic of a serious COVID-19 infection. Because T lymphocyte proliferation depends on telomere length, which decreases with age, elderly patients with COVID-19 are at higher risk for developing telomere length-dependent lymphopenia. Clinical data also indicate that the severity of COVID-19 is associated with limitations in telomere stores in older adults [74]. Thus, manipulation of the telomere/telomerase system may reduce the deleterious effects of aging on lymphocyte function [75], possibly helping to identify high-risk patients and leading to a better therapeutic strategy against SARS-CoV-2 infections [76]. Thus, telomere attrition and dysregulated innate and adaptive immune responses to viral infections also are signs of aging that may contribute to severe consequences of COVID-19 in the elderly.

## 3. ER Stress and the UPR in SARS-CoV-2 Infection

**Figure 2:**
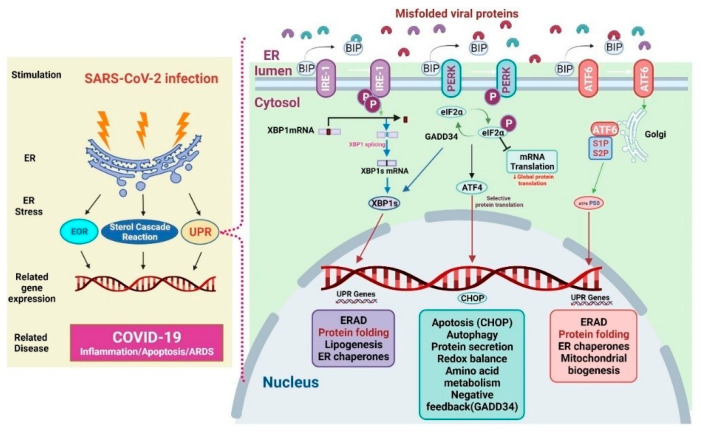
Mechanisms of endoplasmic resticulum (ER) stress and unfolded protein response (UPR) in COVID-19.

The ER represents the largest organelles within the mammalian cell. It is also the major cellular compartment for folding and post-translationally modifying transmembrane and secretory proteins, lipids, and sterols. It also functions as a cellular storage for free calcium [77]. The ER thus accounts for a large proportion of the total protein exported from mammalian cells. When cells undergo viral infection, ER function may change and result in protein accumulation and subsequent ER stress [77]. ER stress initiates downstream gene transcriptional activities and cellular responses such as ER overload response (EOR), UPR, and sterol cascade reaction (SCR), which are aimed at restoring homeostasis. The EOR could be activated by the over-accumulation of properly ER-folded proteins, which further induces a series of cellular signaling pathways. The EOR upon ER stress involves Ca^2+^ release, NF-κB activation, and reactive oxygen species (ROS) production [78]. The EOR works to restore protein processing capacity, oxidative balance, and Ca^2+^ balance in the ER to support cells during stressful periods [79]. The SCR is activated upon the depletion of cholesterol (a key component of cell membranes produced in the ER). Cholesterol depletion may result in proteolysis of the membrane sterol-regulated binding protein (SREBP)-associated transcription factors. SREBP cleavage produces active transcription factors that activate gene expression required for cholesterol and fatty acid absorption and biosynthesis [80,81,82]. It is well known that protein folding is the normal physical process by which proteins acquire their specific spatial structures to perform their specific biological functions. If proteins are improperly unfolded or folded, many abnormal proteins aggregate, triggering ER stress and UPR to restore homeostasis. If these activities are unable to restore homeostasis, various diseases such as diabetes, infectious diseases, cardiovascular diseases, and neurodegenerative diseases can be induced [83] (Figure 2).

ER stress is involved in a series of aging-related diseases. As aging alters physiological stress, protein folding disturbances in the ER that involve the redox balance, Ca^2+^ levels, glycosylation, and other environmental factors, can result in the accumulation of misfolded proteins, leading to ER stress. ER stress triggers an increase in ROS and activates proinflammatory cues and inflammasome formation, indicating that ER stress demonstrates immunogenic effects. ER stress triggers NLRP3 inflammasome activation and the subsequent release of IL-1β. IL-1β is responsible for immune responses to infections and regulates a broad range of proinflammatory activities in immune cells, including the induction of rapid neutrophil recruitment at infection sites, endothelial adhesion molecule activation, and inducing chemokine production. It also induces the secretion of several cytokines, activates the T-helper cells, and promotes B cell proliferation [84]. This ER-triggered proinflammatory signaling has the same requirements as that for ROS production and potassium efflux compared with other known NLRP3 inflammasome activators [85] and can be induced by increased lipid amounts or proinflammatory cytokines [86]. Overall, ER-stress-induced NLRP3 activation induces inflammatory responses by increasing oxidative stress, thereby enhancing the activation of NF-κB and altering the homeostasis of calcium [87].

The UPR is characterized by the presence of the ER-resident chaperones, such as immunoglobulin heavy chain binding protein (BiP, also known as glucose-regulated protein 78 (Grp78)) and glucose-regulated protein 94 (Grp94). Grp78 controls the UPR in the ER of normal cells. Grp78 forces unfolded proteins to fold or degrade using cellular degradation mechanisms [88]. It uses protein kinase R-like ER kinase (PERK), inositol-requiring enzyme 1 (IRE-1), and activating transcription factor 6 (ATF6) as proximal sensors [89,90]. Upon ER stress in COVID-19, Grp78 dissociates from the sensors and binds to unfolded viral proteins, thus activating the sensors and triggering three UPR pathways (Figure 2). The first one is the IRE-1 pathway. IRE-1 exploits an unconventional mRNA splicing manner to induce UPR signaling. ER stress induces autophosphorylation and oligomerization of IRE-1, which is then spliced, and cuts X-box binding protein 1 (XBP1) mRNA to produce truncated XBP1 mRNA that encodes XBP1 in infected cells. The XBP1s induce a set of genes that regulate protein synthesis, ER-associated degradation, protein folding, adipogenesis, and molecular chaperones. The second UPR pathway is the PERK pathway. PERK also undergoes both oligomerization and autophosphorylation to cope with ER stress. Activated PERK will phosphorylate eukaryotic translation initiation factor 2 subunit alpha (eIF2α) and inhibit protein translation. The activated PERK also increases the translation of ATF4 (activating transcription factor 4) selectively, thereby upregulating the genes related to protein secretion, apoptosis, autophagy, and negative feedback mediated by the growth arrest and DNA damage-inducible protein 34 [91]. The third UPR pathway is the ATF6 pathway. In the presence of misfolded proteins, ATF6 moves to the Golgi apparatus and is cleaved by the Site-1 and Site-2 proteases, releasing the transcription factor of ATF6 in its cytoplasmic tail. ATF6 cooperates with XBP1s to increase the transcription of targets that expand ER size and increase its protein-folding capacity, maintaining cell survival through induction of ER-associated degradation (ERAD), protein folding, and mitochondrial biogenesis [10,92].

Mounting evidence implicates ER stress and sustained UPR signaling as major factors for the pathogenesis of viral infections [93]. Responses to invasive viruses are coordinated by PRRs and UPR signaling, which intersect and aggregate viral infection. Although the inflammatory response is intended to be protective during viral infection, it can be too intense or prolonged, thereby increasing disease severity [94,95]. Virus particles can interact with the host UPR to create a favorable environment for virus infections [10]. Although the viral infection induces ER stress [11], ER stress and UPR do not appear likely to defend against reovirus infection but rather to promote replication of the reovirus. Studies have shown that the reovirus replicates most efficiently in the presence of eIF2α kinase, and phosphorylated eIF2α promotes the synthesis of ATF4. Increased ATF4 expression results in a 10- to 100-fold increase in reovirus production [96]. Hepatitis B virus-induced ER stress can elicit the IRE1α/XBP1 signaling pathway, which activates the hepatitis B virus S promoter, leading to enhanced viral production [97].

The PERK pathway aids the host’s defense against the virus [98]. PERK-mediated eIF2α phosphorylation inhibits viral protein synthesis and viral replication [99]. However, phosphorylation of eIF2α fails to induce cell death, or sensitizes cells to killing by pro-apoptotic stimuli. Regulation of eIF2α phosphorylation helps the herpes simplex virus (HSV) survive; HSV promotes ERAD, reduces MHC-I, and suppresses the immune response [100,101]. Human immunodeficiency virus type 1 also uses ERAD and degrades CD4 [101]. The regulated IRE1α-dependent decay (RIDD) can degrade mRNAs to diminish ER stress [102]. ER produces large amounts of membrane proteins and lipids, which are needed for virus replication (Figure 2). Briefly, viral infection may induce ER stress and UPR, inhibiting apoptosis to maintain cell survival. Infection with some viruses will trigger ER-stress-elicited apoptosis by increasing the synthesis of C/EBP homologous protein (CHOP). However, IRE1 can activate RIDD to enhance the degradation of ER mRNAs. Thus, RIDD activation may promote synthesis of viral proteins [10]. However, the CHOP plays a key role in inhibiting RNA virus infection. The porcine circovirus type 2 (PCV2) can enhance the activation of the eIF2α-ATF4-CHOP pathway and caspases, thereby inducing autophagy and apoptotic responses, which may facilitate the PCV2 infection [103]. Induction of eIF2α-CHOP-BCL-2/Jun N-terminal kinases (JNK) and IRE1α-XBP1/JNK signaling cascades promoted apoptosis and cytokine secretion, assisting in the replication of the Newcastle disease virus [104].

In Figure 2, the left panel presents the mechanisms of SARS-CoV-2 infection-induced ER stress. Following ER stress, downstream gene transcription is initiated through the EOR, SCR, and UPR to help restore homeostasis. Improper protein folding and its accumulation lead to increased ER stress and UPR activation [82]. The right panel mainly presents the signaling pathways of UPR. PERK, IRE-1, and ATF6 are proteins that sense ER stress and deliver ER signals to the cytosol. In physiological conditions, Grp78 binds these sensor proteins [105]. ER stress activates the three UPR pathways as follows: (1) During ER stress, IRE1 activates and dissociates from Grp78. After IRE1 activation, it increases XBP1 translation and UPR protein production. (2) ER stress enhances PERK dissociation from Grp78 and PERK phosphorylation; PERK phosphorylation promotes eIF2α phosphorylation, leading to the inhibition of global protein translation and upregulation of ATF4 and CHOP expression. (3) ER stress activates ATF6 dissociation from Grp78. ATF6 is cleaved by S1P/S2P at the Golgi apparatus and the p50 fragment is released. p50 upregulates the expression of XBP1, therefore the expression of UPR-related proteins is also enhanced.

### 3.1. IRE1 and Inflammation

**Figure 3:**
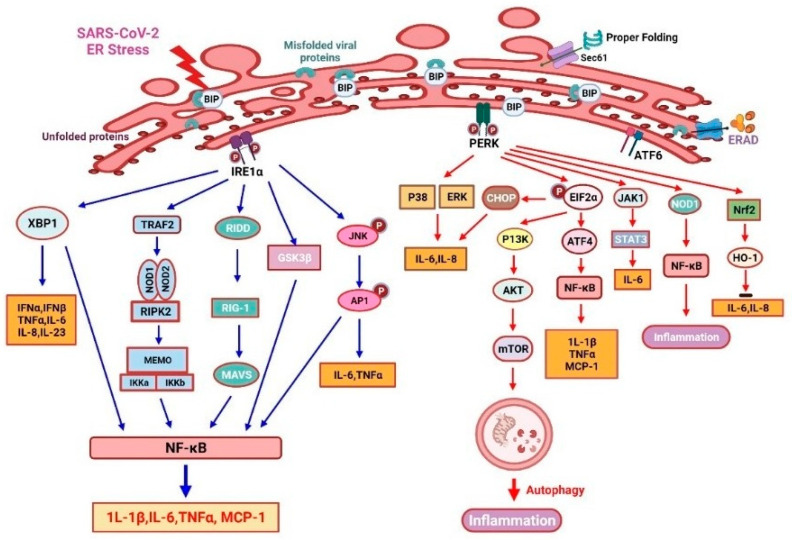
UPR-related inositol-requiring enzyme 1α (IRE-1 α) and protein kinase R-like endoplasmic reticulum kinase (PERK)-mediated inflammation response in COVID-19.

IRE1 is an ER stress sensor that promotes inflammation through several mechanisms (Figure 3). IRE1 modulates inflammatory cytokine secretion, primarily by controlling cellular signals via kinase and endoribonuclease (RNase) activation [106]. The TLR2 and TLR4 may induce IRE1α activation in mouse macrophages upon pathogenic stimuli [107]. IRE1α oligomerization may splice XBP1 mRNA as active transcription factors and RIDD cleaves other mRNAs to induce RIG-1 ligand (an antiviral sensor), triggering an inflammatory response through inside mitochondria MAVS [108]. It also promotes TNF-α secretion from macrophages [109]. The combination complex of IRE1α and TNF receptor-associated factor 2 (TRAF2) subsequently promotes NF-κB inflammasome and JNK- activator protein 1 (AP1) signaling and then activates TNF-α and IL-6 production. The lipopolysaccharide (LPS) induces MCP-1 and other cytokines’ production via the IRE1α/NF-κB pathway, which causes endothelial dysfunction [110]. Furthermore, the IRE1α-mediated activation of GSK-3β inhibits XBP1 splicing and subsequently the transcription of IL-1β, resulting in increased inflammation [111]. Overall, the IRE1 pathway is critical for proinflammatory cytokine production and the efficient elimination of disease-causing viruses or bacteria [67].

### 3.2. PERK Signaling and Inflammation

PERK/eIF2α signaling plays a crucial role in the inflammatory response induced by ER stress (Figure 3). To accentuate the expression of inflammatory genes, the translation blockade by PERK-dependent phosphorylation of eIF2α is essential. The anti-dsDNA antibody can promote NF-κB production and then it upregulates MCP-1 and IL-1β via the PERK-eIF2α-ATF4 signaling pathway in mesangial cells of lupus nephritis [112]. PERK can induce IL-6 secretion via JAK1/STAT3 signaling, then it triggers a feedforward pathway to drive neuroinflammation in autoimmune encephalomyelitis [113]. LPS-treated bronchial epithelial cells recognize PAMPs through the PERK and ATF6/p38/ERK pathways, resulting in the production of various cytokines [114]. Furthermore, thapsigargin (sarco/endoplasmic reticulum Ca^2+^-ATPase inhibitor)-induced PERK activation will sensitize host cells to *Salmonella typhimurium*. Then, thapsigargin will activate an inflammation response and enhance an inositol triphosphate receptor (IPR3)-mediated calcium flux [115]. Thus, pharmacological reprogramming of the stress pathway in ER could be used to suppress SARS-CoV-2 replication. However, as an ER stress inducer, thapsigargin has been shown to effectively diminish SARS-CoV-2 replication in diverse cells, partially reverse virus-induced translation shutdown, increase the survival of infected cells, and counteract SARS-CoV-2-mediated attenuation of IRE1α and BiP expression [14]. Therefore, thapsigargin represents a new prototype of compounds with host-directed antiviral activity based on its mechanism of action [13]. In experimental autoimmune encephalomyelitis (EAE), PERK-eIF2α-induced translational repression will increase the ATF4-independent transcription of CCL20 and IL-6. The inhibition of PERK signaling suppresses inflammation under the ER stress condition [116]. Moreover, PERK phosphorylation induces the expression of its substrate nuclear factor erythroid 2-related factor 2 (Nrf2) to enhance cell survival [117]. Another plant-derived compound, called berberine, negatively modulates inflammatory cytokine secretion by enhancing the PERK/Nrf2/HO-1 signaling, which will ameliorate inflammation [118,119].

As demonstrated in Figure 3, misfolded/unfolded viral proteins activate IRE1α signaling (left), which promotes inflammatory responses. The oligomerization of IRE1α can activate cytoplasmic kinase and endonuclease domains; therefore, augmenting the expression of XBP1s will lead to an increased production of several inflammatory mediators. IRE1 can activate RIG-I through regulated RIDD and trigger a MAVS-related inflammatory response. Furthermore, the IRE1α/TRAF2 complex can enhance JNK-AP1 and NF-κB, as well as TNF-α and IL-6 production. Briefly, eIF2α can upregulate IL-1β and TNF-α by activating the ATF4/NF-κB signaling, while PERK can augment cell viability through the activation of the Nrf2/HO-1 signaling. Studies from human esophageal cancer cells showed that ER stress could induce autophagy by mediating the PI3K/Akt/mTOR signaling pathway [120]. PERK/eIF2α signaling will enhance autophagy by activating the PI3K/AKT/mTOR signaling pathway, which may also help to suppress cellular inflammation. PERK/eIF2α signaling also suppresses translation and consequently enhances the transcription of various chemokines and cytokines [104]. PERK can enhance NF-κB expression and regulate JAK1/STAT3 signaling in a NOD1-dependent way to increase IL-6 secretion. In addition, the PERK/p38/ERK signaling axis will favor the production of IL-8 and IL-6 [113].

### 3.3. ATF6 and Inflammation

The two ATF6 paralogs and five CREB3 proteins are members of the ATF6 family. Among the ATF6 members, ATF6α still responds effectively in disease-induced ER stress, even without ATF6β, whereas the response of ATF6β is diminished without ATF6α [121]. The ATF6 response during inflammation is often overlooked; this situation is unlike the other UPR pathways and requires further studies [119,122].

### 3.4. NLRP3 Inflammasome Involvement in ER Stress Elicits an Inflammatory Response

The NLRP3 inflammasome includes intracellular innate immune receptors NLRP3, apoptosis-associated speck-like protein (ASC), and caspase 1. It recognizes DAMPs or PAMPs and induces protease caspase-1 activation [123]. The activated caspase-1 cleaves IL-1β and IL-18 precursors into their mature forms. Multiple endogenous and exogenous stimuli, including virus infections, LPS, and crystalline deposits, can activate the NLRP3 inflammasome. The NLRP3 inflammasome participates in calcium ion imbalance, lysosomal damage, potassium outflow, and ROS generation [124,125].

**Figure 4:**
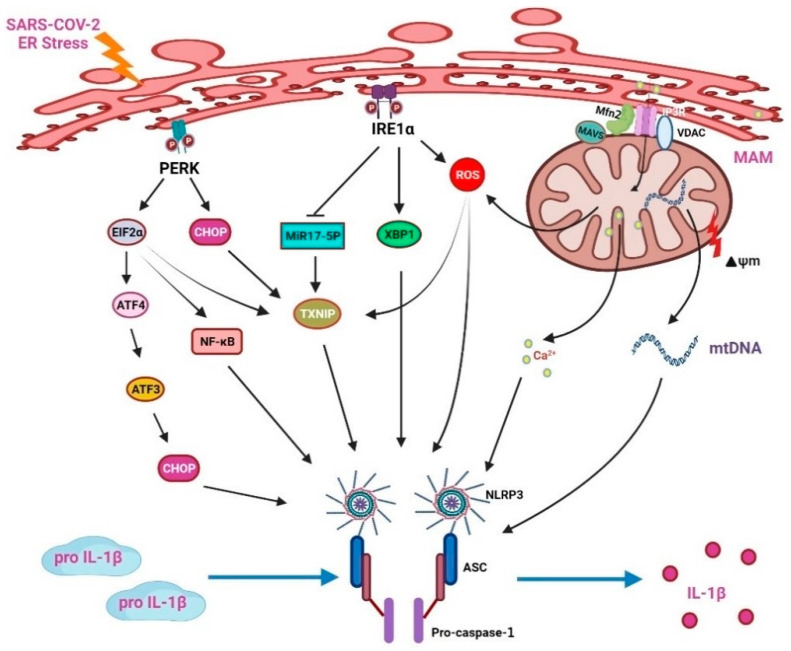
NOD-like receptor protein 3 (NLRP3) inflammasomes mediate ER stress-induced inflammatory responses.

ER stress is also related to the activation of the NLRP3 inflammasome (Figure 4). IRE1α activation enhances ROS levels in the mitochondria and strengthens the association between mitochondria and NLRP3. NLRP3/caspase-2-induced mitochondrial ROS production and ER stress will promote inflammation, thereby presenting with the characteristics of ER stress and innate immunity [126]. Under ER stress, the IRE1 can enhance ROS production; then, the NLRP3 inflammasomes accumulate to aggravate IL-1β maturation in B cells [127,128]. In diabetic rats, IRE1α can upregulate miR-200a degradation and then stimulate the TXINP/NLRP3 pathway-mediated pyroptosis [129]. In pancreatic β cells, NLRP3 inflammasome production can be increased by IRE1α and PERK/eIF2α signaling activation of the thioredoxin interacting protein (TXNIP)/NLRP3 pathway [130,131]. The ER releases Ca^2+^ during stress, which induces ROS production in mitochondria and enhances the activation of NLRP3. The ATF3 signaling pathway also upregulates CHOP expression to enhance IL-23p19 production [132]. The CHOP will fasten the opening of voltage-dependent anion channels (VDAC) on the mitochondrial outer membrane [33,133]. Blocking VDAC reduces NLRP3 inflammasome assembly and then suppresses inflammatory cytokine production [134].

As demonstrated in Figure 4, the ER stress-induced NLRP3 inflammasome production is the crucial mechanism of the inflammatory reaction. PERK phosphorylates eIF2α, thereby partly promoting the activation of downstream NF-κB and TXNIP. However, activation of the PERK/eIF2α/ATF3 signaling pathway will enhance the production of CHOP. In addition, the CHOP will promote the opening of VDAC to release Ca^2+^ from the mitochondria which will in turn activate the NLRP3 inflammasomes and release various cytokines, such as IL-1β. A previous study also showed that the ER stress inhibitor blocks LPS-induced liver inflammation through the downregulation of CHOP, reduces various caspase activities, and decreases IL-1β secretion [135]. ER stress increases the production of ROS. The ROS will dissociate TXNIP from thioredoxin (TRX), allowing TXNIP to bind to NLRP3 and leading to the activation of the NLRP3 inflammasomes. During stress, Ca^2+^ ions that are accumulated in the ER will be delivered to the mitochondria by the mitochondrial-associated membrane (MAM), which will further increase the degree of oxidative stress and mtDNA injury.

### 3.5. HLA Classes I and II, ER, and SARS-CoV-2 Infection

ER stress and complications/disorders of the immune response are associated with the SARS-CoV-2 infection. The processing, presentation, and assembly of human leukocyte antigen (HLA) classes I and II with the viral antigens take place within the ER. Kesheh et al. reviewed bioinformatic studies for HLA alleles associated with preexisting conditions, including cardiovascular disease, obesity, diabetes mellitus, chronic obstructive pulmonary disorder (COPD), and chronic kidney disease, that exacerbate the SARS-CoV-2 infection [136]. Recently, Shira et al. found new HLA-I peptides originating from out-of-frame ORFs that elicit strong T cell responses, thereby representing the important role of HLA [137]. Tomita et al. used an in silico analysis for HLA gene polymorphisms of confirmed COVID-19 and deaths from 19 countries. Furthermore, they found that HLA-A*02:01 variants decreased the SARS-CoV-2 antigen presentation and increased the poor outcome of COVID-19. Furthermore, they found HLA-A*11:01 and HLA-A*24:02 variants have a more efficient T cell-mediated immune response to SARS-CoV-2 than HLA-A*02:01 variants. [138]. Shkurnikov et al. studied HLA class I genotypes in patients with varying severities of COVID-19 in Russia; the blood samples from 111 deceased COVID-19 patients and 428 volunteers were examined with next-generation sequencing. They found that the HLA-A*01:01 allele was associated with a high risk of severe COVID-19 [139]. Vietzen et al. studied gene variants in 361 patients with mild to severe cases of COVID-19 in Austria and found that *KLRC2* deletion combined with HLA-E*0101 predominant distribution in ICU COVID-19 patients characterized old age and more comorbidities [140]. Weiner et al. conducted a multicenter study on HLAs and COVID-19 severity in 435 individuals from Germany, Spain, Switzerland, and the United States [141]. They found that carriers of HLA-C*04:01 have a severe clinical course of COVID-19 and are at twice the risk of intubation for SARS-CoV-2 infection than non-carriers. Recently, Yoo et al. found that the SARS-CoV-2 ORF6 protein suppresses the MHC class I transactivator NLRC5. Francis et al. found that the HLA class I allele variation may influence the CD8+ T cell repertoire shape and immune recall to the SARS-CoV-2 infection [142]. Gutiérrez-Bautista et al. studied 87 individuals who were vaccinated with the mRNA-1273 vaccine and 637 healthy blood donors as the control group in Spain [143]. They found that certain HLA-class II alleles, such as HLA-DRB1*07:01, have a higher production of antibodies on the 30th day after the second dose of mRNA-1273. HLA plays an important role in genetic susceptibility to human infectious diseases similar to COVID-19 [144]. However, HLA from the ER and the association thereof with age and comorbidities of the SARS-CoV-2 infection still requires further study.

## 4. Melatonin and COVID-19

Anderson et al. reviewed the pathophysiology of aryl hydrocarbon receptors (AhR) during viral infections [145]. During SARS-CoV-2 infection, comorbidities such as aging, type 2 diabetes, and obesity increase the production of pro-inflammatory cytokines and activate the AhR. The activation of AhR combined with decreased melatonin during influenza and SARS-CoV-2 infections upregulates the activation of immune cells and the exhaustion of natural killer cells and CD8 T lymphocytes. Previous comorbidities in patients with influenza A infection such as cardiovascular, pulmonary disease, diabetes, and cancer increase mortality risk [146]. In the other reviews influenza infection, increased miRNAs inhibit 14-3-3 and suppress the melatonergic pathway [147]. The circadian gene, *Bmal1* that regulate the mitochondrial melatonergic pathway is inhibited during influenza infection. Upon addition of melatonin, it promoted *Bmal1* activity on the mitochondrial melatonergic pathway and inhibited viral replication. This pathophysiology feature finally determines the severity of SARS-CoV-2 and influenza infections. Moreover, it highlights the importance of mitochondrial melatonergic pathway regulation.

### 4.1. Melatonin, Viral Infection, and ER Stress

Viral infections have been associated with ER stress and there is increasing research attention on the components of the UPR as therapeutic strategies. Melatonin can regulate ER stress and augment the UPR response during various viral infections. It also exerts anti-inflammatory and antioxidant effects; therefore, it regulates apoptosis, autophagy, neurodegeneration, and other pathologies in cancer cells [148]. Melatonin also reduces macrophage inflammation by modulating a series of signaling pathways related to ER stress [149]. In rabbit hemorrhagic disease virus (RHDV) infection, treatment with melatonin decreases immunoreactivity for the viral VP60 antigen and reduces the activity of caspase-3 and levels of BiP and CHOP expressions. These results suggest that melatonin can induce a reduction in RHDV infection-related autophagy and inhibit RHDV RNA replication [150].

Melatonin inhibits mammalian sterile 20-like kinases 1 (Mst1) and ameliorates viral myocarditis by improving cardiomyocyte viability and attenuating both ER stress and mitochondrial damage, as indicated by an improvement in decreased mitochondrial membrane potential and increased mPTP opening and activation of caspase-9-associated apoptosis [151]. In an in vitro colorectal cancer (CRC) cell model, melatonin treatment demonstrated significantly enhanced autophagy through the ER stress pathway [152]. Melatonin can effectively activate the intracellular antioxidant TRX-1, markedly inhibiting the TXNIP/NLRP3 pathway and inhibiting an abnormal activation of mitophagy-induced inflammation. Melatonin also improved the overall antioxidant state of the lungs with COPD by restoring the NRF-2-HO-1 axis [153]. In a murine model of testicular hyperthermia, both the ER stress and testes apoptosis were induced by heat stress. Pretreatment with melatonin inhibited ATF6 and PERK, resulting in heat-induced apoptosis. This relief was dependent on melatonin receptors [154]. As an ROS scavenger and an inducer of antioxidants in both in vivo and in vitro studies, melatonin exhibits antioxidant properties [155]. However, a recent study reported the pro-oxidant activity of melatonin when used at high concentrations [156]. In cancer cells, melatonin showed pro-apoptotic effects, whereas it was present with anti-apoptotic effects in normal cells [157]. Since oxidative stress contributes to UPR activation, melatonin may exert its protective effects on cells by modulating the ER stress response through its oxidative–anti-oxidative properties [158].

### 4.2. Melatonin Modulates Nrf2 and Related Signaling Pathways

Nrf2 is a well-known sensor of oxidative stress. The Nrf2 signaling pathway maintains physiological conditions via redox balance. Various medicines have been developed to reduce oxidative stress in clinical diseases. Naturally occurring compounds are of particular interest because of their valuable biological activities and minimal side effects. Numerous studies have shown that melatonin has multiple beneficial effects such as antioxidant, anti-inflammatory, anti-tumor, anti-diabetic, and cardio-protective effects [159]. The anti-senescence effects of melatonin also result from ER stress inhibition by modulating the Nrf2 signaling pathway.

### 4.3. Melatonin Adjuvant Treatment for COVID-19

Melatonin is well known to possess antioxidative, anti-inflammatory, and immunomodulatory effects on viral diseases, such as RSV, influenza A, and Ebola, rendering it a potential therapeutic candidate for early COVID-19 treatment [160].

In the course of the pandemic, many clinical studies, with different outcomes, were performed. A large multicenter retrospective observational study conducted in France found that of a total of 58,562 hospitalized COVID-19 patients (suspected mild to critical severity), only 272 patients received variable dosages of melatonin (mean daily dose of 2.61 mg) for a mean duration of 15.6 days [161]. There was no significant benefit of melatonin for survival, which thus merits a large prospective randomized control trial in the future. Lan et al. performed a meta-analysis based on three randomized control trials [162,163,164] that administered melatonin to patients with COVID-19. They found that oral administration of melatonin 3–9 mg daily for 7–14 days increased the clinical recovery rate, lowered the intensive care unit admission rate, and lowered the mortality rate compared with those in the placebo group [165].

In a pilot study conducted on the quality of life of patients in the US with mild to moderate COVID-19 severity, 32 patients who were supplemented with 10 mg of melatonin daily for 14 days had a more rapid improvement in their quality of life than the placebo group of 34 patients [166]. In a randomized single-blind pilot study of Iranian patients with mild to moderate COVID-19 severity, 14 patients received 6 mg of melatonin for 2 weeks. In the patient group, it was found that there was an inprovement in the C-reactive protein (CRP) level and in recovery from symptoms, compared with that in the 17 control patients. However, this was not considered significant because of the small sample sizes [162]. In a single-center double-blind randomized clinical trial of hospitalized patients with mild to moderate COVID-19 severity in Iran, 24 patients in the intervention group received 9 mg of melatonin daily for 14 days and 20 patients in the control group received standard therapy. It was found that the intervention group had considerable improvement of clinical symptoms and CRP compared with the control group. In another clinical study of hospitalized Iranian patients with mild to moderate COVID-19 severity, 20 patients received melatonin supplement (9 mg daily for 14 days) and 20 patients formed the control group [167]. The melatonin supplement group had a significant reduction in TNF- α, IL-1β, malondialdehyde, and nitric oxide and an increased level of superoxide dismutase compared with the control group.

In a prospective randomized clinical trial of severe COVID-19 in Iraq, 82 patients were administered melatonin (standard therapy plus 10 mg melatonin daily for two weeks), while 76 patients were provided standard therapy only; the melatonin-treated group presented less thrombosis and sepsis events after adjusting for other confounding factors and lower mortality (melatonin group vs. control group: 1.2% vs. 17.1%) [168]. In another randomized controlled trial of melatonin treatment for hospitalized Iranian patients with severe COVID-19, a total of 109 patients in the melatonin group (adjuvant 5 mg melatonin twice daily for 7 days) and 117 patients in the control group (standard therapy only) were enrolled in the study [169]. The mortality rate was 67% in the melatonin group compared with 94% in the control group. Because it was a randomized control trial, the results were not adjusted for other confounding factors but did show that the melatonin group had a lower in-hospital mortality rate, lower invasive mechanical ventilation use, shorter hospitalization duration, and better clinical status than the control groups. Furthermore, a large retrospective observational study enrolled 791 critical COVID-19 intubated patients with a total of 948 intubation periods from the New York Presbyterian/Columbia University Irving Medical Center. Statistical analysis with Cox proportional hazard ratios for the COVID-19 intubation periods showed that melatonin exposure (with varied dosages) resulted in a significant advantage for the survival of patients who had undergone mechanical ventilation (*p* < 0.01) [170]. In another study, high-dose melatonin adjuvant therapy in critical intubated COVID-19 patients study showed that 33 patients administered with melatonin (21 mg daily for 5 days) had no significant benefit in mortality than the 34 patients of the control group [171]; however CRP was significantly decreased in the melatonin group than in the control group (*p* < 0.05).

In an open-label randomized controlled COVID-19 sleep-quality study conducted in Iran, 48 patients were enrolled in the melatonin group (3 mg daily for 7 days) and 48 in the control (standard therapy) group [164]. The melatonin group had remarkably better sleep quality and blood oxygen saturation than the control group. In a retrospective observational sleep-quality study on non-invasive ventilation for COVID-19 patients conducted in Italy, 40 patients in the melatonin group (2 mg daily for the duration of the patients’ stay in the sub-intensive care unit) experienced considerably longer sleep hours and decreased delirium episodes compared with the control group [172]. Furthermore, patients infected with SARS-CoV-2 may have prolonged symptoms of cognitive impairment, fatigue, and respiratory and metabolic system abnormalities for six months to two years following infection, implying long-COVID syndrome. Melatonin may have potential therapeutic benefits on myalgic encephalomyelitis and chronic fatigue syndrome, but this still needs further prospective study [173]. Overall, melatonin is a promising potential therapeutic agent for COVID-19 cases with mild to critical severity, but further prospective multicenter randomized control trials concerning age and comorbidities are needed.

## 5. Conclusions

COVID-19 continues to threaten human life worldwide and can cause repeated pandemics. Although vaccines and current antiviral therapies provide relief from critical COVID-19, the mortality rates are still alarming in some countries, thus additive therapy is urgent. Many recent studies have suggested the potential of melatonin in treating COVID-19, owing to its multifaceted beneficial effects. Elderly people with comorbidities are particularly susceptible to COVID-19 and aging is associated with immunosenescence, inflammation, inflammasome formation, and altered autophagy. Aging leads to ER stress, disturbed protein folding, improper redox balance, and misfolded protein accumulation. Since melatonin is an age-dependent chemical, older people have low levels of melatonin. As SARS-CoV-2 infection can enhance inflammasome formation and inflammation, melatonin may be benefitial in reducing these inflammatory complications, such as pulmonary fibrosis. Melatonin may also modulate ER stress and UPR stimulated by viral infections.

Based on the above information, melatonin has the potential to ameliorate inflammation and inhibit SARS-CoV-2-induced cytokine storms. Melatonin is recommended for the early treatment of infection, as it stimulates a suppressed immune system and, in the case of inflammation, it exerts an immunosuppressive effect. In COVID-19, melatonin may reduce long-term inflammation and oxidative effects of the virus, thus allowing the patient’s immune system to respond appropriately to the infection and possibly recover quickly. Although the side effects of melatonin remain relatively benign, further prospective clinical studies should be conducted to confirm the benefits of melatonin in elderly patients with COVID-19.

## Data Availability

This is a narrative review article. The primary collection of documents for analysis and review comes from PubMed.

## Abbreviations

ACE—angiotensin-converting enzyme; Akt—protein kinase B; AP1—activated protein 1; ATF3—activating transcription factor 3; ATF4—activating transcription factor 4; CAT—catalase; CHOP—C/EBP homologous protein; COX-2—cyclooxygenase-2; eIF2α—eukaryotic translation initiation factor 2α; ER—endoplasmic reticulum; ERK—extracellular signal-regulated kinase; Gas6—growth arrest-specific 6; GSH—glutathione peroxidase; GSK-3β—glycogen synthase kinase-3β; HMGB1—high-mobility group box-1; HO-1—heme oxygenase-1; IL—interleukin; iNOS—inducible nitric oxide synthase; IRE1—inositol requiring enzyme 1; JAK1—Janus kinase 1; JNK—c-Jun aminoterminal kinase; MAM—mitochondrial-associated membrane, apoptosis-associated speck-like protein (ASC); MAVS—mitochondrial antiviral signaling protein; MDA—malondialdehyde; mTOR—mammalian target of rapamycin; mTORC1—mechanistic target of rapamycin receptor complex 1; NF-jB—nuclear factor-jB; NF-κB—nuclear factor κB; NICD—intracellular domain of Notch; NLRP3—NLR family pyrin domain-containing 3; NOD1—nucleotide-oligomerization domain-containing proteins 1; Nrf2—nuclear factor E2-related factor 2; PERK—protein kinase RNA (PKR-)-like kinase; PI3K—phosphatidylinositol 3-kinase; PKC—protein kinase C; RIDD—IRE1-dependent decay; RIG-I—retinoic acid–inducible gene I; RIPK1—receptor-interacting protein kinase 1; ROS—reactive oxygen species; SIRT—sirtuin; SOD—superoxide dismutase; STAT3—signal transducer and activator of transcription 3; TLR4—toll-like receptor 4; TNF-α—tumor necrosis factor-α; TRAF2—TNF receptor-related factor 2; TXNIP—thioredoxin-interacting protein; VDAC—voltage-dependent anion channels; XBP1—X-box binding protein 1.
